# Engaging People in Making History: Impact, Public Engagement and the World Beyond the Campus

**DOI:** 10.1093/hwj/dbv015

**Published:** 2015-09-23

**Authors:** Laura King, Gary Rivett

**Affiliations:** University of Leeds; York St John University

## Abstract

By examining the longer history of engagement between academics and those outside the academy and reflecting on recent experiences of collaboration, this paper provides a critical perspective on understandings of engagement and the ‘impact’ of historical research today. Considering in particular the UK higher education landscape and the recent Research Excellence Framework measurement exercise, we argue that the current approach of universities, and understandings of the relationship between them and those outside higher education, promotes a model of one-way dissemination, entails a potentially paternalistic approach to an apparently passive public, and favours easily measurable change. We suggest that by revisiting the intellectual origins of the public-history movement we can better understand where the value in the relationship between academics and the public lies. Our conclusion is that refocusing on the process of engagement – rather than specific and easily evaluated outcomes – better reflects and values the most successful, productive and democratic collaborations between researchers and non-academic partners.

## ***

Universities and academics constantly find new and exciting ways to engage with the world beyond the campus. The idea of working in genuine collaboration with non-academics is not new. Since the 1960s and earlier, the history from below and oral history movements have involved community groups and members in the process of knowledge production. The History Workshop movement of the late 1960s and the intellectual contribution of Raphael Samuel were particularly important in expanding definitions of authority and ownership over the past. By the 1990s, Samuel was observing that ‘history is not the prerogative of the historian, nor even, as postmodernism contends, a historian’s invention. It is, rather, a social form of knowledge; the work, in any given instance, of a thousand different hands’.^1^ The intellectual rationale behind such external engagement has expressed itself through a growing literature on public history, though the concept has never had a fixed definition.^2^ This article suggests that the ideas of history-from-below thinkers such as Samuel remain important and can be used to criticize current instrumentalist ideas about the uses of history, in the UK notably the notion of ‘impact’. As Samuel noted in 1981, people’s history helped to challenge ‘professionalized monopolies of knowledge’. This is again an important aim within the current ‘impact agenda’ which prioritizes one-way dissemination from university to public. Our own experiences of engagement, collaboration and maximizing research impact, as modern (King) and early modern (Rivett) British historians, are indicated by our title. Emphasis on the process of making histories has proved particularly successful in our own work with non-academic partners. We have both focused on contemporary issues and histories in our engagement, but it is most certainly possible to bring in historical knowledge from a wide range of periods and places.^3^ In this article, we draw on our own experiences, in particular a collaboration with a theatre company, Babakas, around the theme of fatherhood, and the Sheffield Stories of Activism project. Here we offer some balanced criticism of the ways in which universities conceptualize their relationship with partners and publics beyond the campus, considering the current context of higher education in the UK in particular.

Historians in recent years have been reflecting on how history can be useful. Some suggest the arts and humanities have an innate value and draw, knowingly or not, upon a venerated tradition in which universities exist as a protected space wherein the highest of ‘aspirations and ideals’ can be reached.^4^ Others have articulated the ways in which history has a role in the present and future. As Pat Thane wrote in 2009, history is not just about heritage or a distant and non-useful past, but can be of immediate use in the present, not least to politicians.^5^ Pam Cox expanded this discussion in 2013,^6^ while in *The History Manifesto* (2014) Jo Guldi and David Armitage brought these debates to a broader audience, and claimed that historians’ unique training can contribute to finding solutions to the most urgent contemporary problems: inequality, a crisis of global governance and climate change.^7^ Their call for historians to participate more actively in public life has been widely debated and criticized, not least because it overlooks important examples of how historians are already engaging with, for example, policymakers.^8^

More recently, funding for co-produced research has become increasingly common, and in the UK, the Arts and Humanities Research Council’s (AHRC) recent ‘Connected Communities’ scheme has demonstrated the many ways in which academic historians can effectively work in collaboration.^9^ The UK National Co-ordinating Centre for Public Engagement aims to promote a wide range of engagement activity, and encourages universities to sign up to its manifesto, that institutions should share ‘our knowledge, resources and skills with the public’, but also learn from ‘the expertise and insight of the different communities with which we engage’.^10^ Importantly, though, these developments have a broader history that precedes recent imperatives and debates concerning impact. The University of Birmingham’s Centre for Contemporary Cultural Studies (1964–2002) pioneered partnerships with educational institutions, and aimed to have an impact in the immediate locale. In London the Raphael Samuel Centre was set up in 1995 with the aim of ‘encouraging the widest possible participation in historical research and debate’, and creates local-historical initiatives in collaboration with local history organizations.^11^ Working with non-professional historians, in ways which value their expertise, is not new.

Yet academics who collaborate beyond the academy now do so in a framework shaped increasingly by the concepts of ‘public engagement’ and – more importantly in the UK – ‘impact’. Funding applications have to illustrate ‘Pathways to Impact’ for the Research Council United Kingdom (RCUK) and to demonstrate measurable research impact in the recent Research Excellence Framework (REF) exercise. Because of such requirements relationships between academics and their partners and audiences beyond academia are pushed into central debates about universities’ missions and the priorities of staff members. The impetus to work with partners outside of the university and members of the public is a good thing; nevertheless, current conceptions of ‘impact’ are problematic. The term implies one-way dissemination, a paternalistic approach to a passive public, and an easily measurable phenomenon. Historians can struggle, however, to demonstrate how their particular research has had an impact, and to evaluate the relative extent of that impact, particularly for REF documents. Yet, even when research-council definitions of impact allow for a rather broader approach, much of the most ‘impactful’ and significant work of historians engaging beyond the campus falls outside the current definition of impact, and is not captured or valued.

According to one early post-REF analysis, sub-panels were conscious of differences between disciplines and recognized that impact was not always comparable across them.^12^ Nonetheless the conception of impact in the UK remains too focused on the outputs and endpoints of engagement activities, rather than valuing a process of two-way engagement or valuing the expertise of non-academics as Samuel suggested. The need to prove one-way impact has led to a constraining model of engagement that does not take account of valuable practices, such as when members of the public are research informants or act as voluntary researchers, or when partner organizations shape research questions. In this article we challenge that top-down model and instead propose one which works from the bottom up and emphasizes the processes and relationships involved in engagement between academics and external publics and partners, as part of a longer history which values the input and expertise of a wide range of participants.

We are certainly not suggesting that every academic, university department or external-relations officer agree with REF-driven criteria for impact. Nor do we want to imply that they have all slavishly followed the guidelines without creative deviation or outright challenge. Numbers of creative individuals and groups have worked at the edges of what might be considered good impact practice and by so doing have problematized the impact agenda, and its emphasis on outputs and outcomes. We intend to give some shape to the problems encountered by the historians working at the ‘coal face’ of impact and engagement, and those who support them. All too often engagement activities can slide towards ‘tick box’ exercises, which have to be ‘top-down’ in order to fulfil the needs of universities. If academics ‘do’ engagement and/or impact work because it is necessary to secure funding or enhance promotion opportunities,^13^ or if doctoral students and early career researchers ‘do’ it because it enhances career prospects, who benefits from their research? May our efforts actually reproduce and reinforce intellectual boundaries, and thereby sustain divisions between academics and the public? These questions emerge from a commitment to Samuel’s position on the nature of historical knowledge.

## A CRITIQUE OF IMPACT

The last Research Assessment Exercise (RAE) was held in 2008 and in 2014 a new system took over: the Research Excellence Framework. ‘Impact’ had meanwhile become one of the criteria for judging the value of research in the UK. When the RAE was introduced, in 1986, the aim was to evaluate research quality in UK universities and funding was allocated by the University Grants Committee according to the results. The inclusion of ‘impact’ as a criterion of assessment by the Higher Education Funding Council for England (HEFCE) marked a substantial addition to the ostensibly new REF. REF panels assessed three areas. Research output – peer-reviewed publications – made up sixty-five percent of the research quality rating, with research environment, focused on grant income and research students’ success, accounting for a further fifteen percent. The remaining twenty percent (which rose to twenty-five percent in future exercises) came from the economic and social benefits beyond the academy that were a direct result of excellent research – or ‘impact’.^14^

The Research Councils and the REF define impact differently. Both conceive it as underpinned by excellent research, but while the RCUK is a funding body and therefore, at least in theory, receives applications for projects which will result in outputs or outcomes that as yet do not exist, the REF is an assessment exercise, which evaluates retrospectively the quality of research outputs and outcomes. As a result, the approach to framing impact is, necessarily, different.^15^ The RCUK recognizes that impact can emerge at different points of the research process. Its guidelines state that the ‘Impact summaries and Pathways to Impact are designed to encourage researchers to start thinking about potential beneficiaries … while planning the project’.^16^ This has the potential to encourage a dialogue with partners and other beneficiaries whilst the research project is in a planning stage. Furthermore, by its provision of ‘follow on’ funding for research, which lets researchers apply for separate funding to support unanticipated impact, RCUK acknowledges that the eventual impact of research is unpredictable.^17^

The REF has a different emphasis. It requires impact case studies which demonstrate the link between particular research findings and observable and provable impact. Case studies contain narratives (with evidence) which demonstrate ‘how the research underpinned (made distinct and material contribution to) the impact’ and ‘the nature and extent of the impact’. Evidence can take the form of quantitative indicators, critiques or citations in users’ documents, independent testimony and formal evaluations. Throughout the extensive guidance for reporting impact, it is clear that the ‘underpinning research’ should be completed. That research needs to be of a certain quality, and judgement should be derived from the usual peer-review processes, end-of-grant reviews and so on.^18^ In the 2014 REF impact was judged by ‘reach’ and ‘significance’, roughly correlating with quantitative and qualitative assessment methods, respectively. The emphasis is on completed, excellent research which leads to a change; the Panel assessed ‘the degree to which the impact enriched, influenced, informed or changed the policies, practices, understandings or awareness of organizations, communities or individuals’.^19^ This system potentially rewards clearly defined and measurable linear change, in cases where a piece of published research led to a measurable impact. However, the influence a researcher might have early on in their research process – when more meaningful engagement can occur – may be overlooked.

Understandable though this shift may be – the RCUK and REF are located at different points of the research spectrum – the different understandings of impact have had implications for how historians have engaged with the impact agenda. Over the last three years the REF has pushed definitions of impact towards more fixed ideas about achieving certain outcomes and end points rather than exploring the multitude of ways in which the research might be of use or of interest to varying groups and individuals outside the academy. One fear is that the outcomes of the REF might further this process, with university departments looking to replicate highly-rated case studies and examples of impact, or to devise careful submission strategies to manipulate the process instead of focusing on meaningful collaboration and exchange.^20^ The timescales set by REF measurements also disadvantage research whose impact might be much more long-term, as well as early-career researchers or others moving between institutions. Because the published research and impact need to have happened at the same institution, the work of scholars who move between universities or even departments may not be recognized as valuable. REF criteria therefore value only a certain section of excellent engagement with non-academic bodies and individuals.

In the link to particular published research findings, current REF definitions of impact could also exclude the wider influence that a historian can have on those they work with, for instance in rethinking important concepts, in enhancing the skills of those outside the academy, and in critiquing notions of what is natural, timeless, unchanging or new within today’s society. Such influence does not necessarily have roots in our articles and books, but in our training and expertise as historians. The AHRC and Heritage Lottery Fund ‘All Our Stories’ projects privileged such an approach: they focused on ‘community-led community heritage research projects’, which involved academics but were not directed by them whether in subject matter, research questions or ways of working.^21^ Despite statements that the REF would consider how ‘staff … interacted with, engaged with or developed relationships’ with the public, it remained unclear how interactions would be appraised.^22^ Thus, the assessment of our impact as researchers – rather than the impact of our specific research findings – remained vague. It may be that it is in fact impossible for REF reviewers to distinguish clearly between impact from the research findings and from the process itself. However, a broadening out of what might constitute the acceptable origin of the impact – the research specifically, the research process and/or the researchers themselves – would be a welcome change. Appreciation of the importance of collaborative relationships to successful research impact has precedents. The CASE industrial studentship scheme created in the 1990s and Collaborative Doctoral Awards (CDAs) that partner scholars and doctoral students with non-academic organizations point to the value of co-production. (CDAs or indeed impact from any PhD students are not currently included in how REF measures impact.) Recognizing the process of engagement would, ultimately, better reflect and recognize the potential value of arts and humanities research, broadly conceived.

RCUK funding requirements and REF impact case studies are, of course, part of a wider context of governmental interventions in academic life. However, the techniques of these interventions have received little attention. Our second critique suggests that the impact agenda is informed by a kind of ‘soft paternalism’, which instrumentalizes two-way engagement for the purposes of ‘impact’. Soft paternalism represents an ‘overt process through which subjects are encouraged and, indeed, actively buy into particular kinds of behaviour to improve their own (and others’) welfare’.^23^ Soft paternalism is a concept that is related to a variety of governmental policy areas and is most commonly associated with the idea of ‘nudging’: the gradual and directed attempt by governors to transform behavioural practices.^24^

Paternalistic and top-down approaches to engaging beyond the campus naturally value outcomes over processes. First, the discourse around impact is revealing about the role of the academic and the public. The outcomes and longer-term impacts of research should seek to ensure the public is ‘empowered’, has its ‘attitudes broadened’, is ‘inspired’. Academics should endeavour to achieve these outcomes because, as the RCUK’s keynote document, the *Concordat for Engaging the Public with Research* (2010) states, ‘Public Engagement can … help universities actively contribute to positive change and the ‘public good’.^25^ Whilst none of these aims are inherently bad, the formulation reaffirms a hierarchical model in which an academic is placed above ‘the public’ as a whole. The language surrounding impact, outputs and outcomes suggests a set of preferences for certain kinds of socially acceptable citizens. Somewhat paradoxically, such change in attitudes though apparently valued in REF terminology is near impossible to measure according to its criteria.

This soft paternalism encourages universities to institutionalize public engagement. As an extension of the Department of Business, Innovation and Skills (BIS), UK universities and academics are instrumentalized by governments. The RCUK’s subsequent *Inspiration to Engage: Concordat for Engaging the Public with Research* (2013) demonstrates how government promotes and directs the priorities of public engagement by providing incentives for academics to conduct public engagement based upon their research. According to the principles ‘drawn up by the funders of research in the UK’, ‘public engagement thrives when there is a strategic commitment “from the top”’.^26^ The document appears to offer guidance on good practice for public engagement, but is more accurately a manifesto for why academics should engage with the public, advertising how public engagement can enhance the career prospects of entrepreneurial academics. University training, professional development, and review processes will ensure that public engagement is correctly pursued, funded and monitored (the REF is one such monitoring technique).^27^ Conversely, *Inspiration to Engage* is vague on what role the public plays in the process of engagement, and how people beyond the campus benefit from research. In case studies presented as evidence of good practice, it is notable how the public is of use to the researcher. The public therefore gains some abstract benefit and is instrumentalized as a part of BIS’s broader priorities, with the force of direction emanating from above.^28^

Funding proposals and assessment exercises, incentives for academics to engage the public with their research, the impact agenda in the UK, and, in particular, the current methods for judging the quality of public-engagement activities based on outcomes, all emphasize top-down governmental priorities. Furthermore, by the terms of current assessment criteria it is assumed that research that has an impact will be welcomed by the public. Advocates of public engagement make similar presumptions. However, as Stefan Collini has noted, the REF ‘proposes no way of judging whether an impact is desirable; it assumes that if the research in question can be shown to have affected a number of people who are categorized as outside, then it constitutes a social benefit of that research’.^29^ In short, what about a highly significant impact from academic research that is in some way harmful? None of this is intended to suggest that academics and universities should not be involved in inspiring, empowering and educating the people with whom they engage where this is appropriate. Nor is it to disagree with the notion that governments, as representatives of the electorate, should have a say in how public funds should be distributed. Nonetheless, paternalism of the kind described here has the potential to reinforce distinctions between universities and the public. If universities are perceived as doing something ‘for’ the public, without the public being involved in determining what might be of benefit to, say, a local community, higher-education institutions have the potential to be another kind of service-provider: an institution that is arms-length, undemocratic and dispenses knowledge from afar.

Therefore, to suggest that the impact agenda is softly paternalist draws attention to a set of priorities that need to be carefully considered when working with the public, or disseminating research: what are we actually doing? Are the currently defined research ‘impacts’, ‘outputs’ and ‘outcomes’ the most appropriate ways to shape the relationship between research and engagement? What role should individuals and other organizations have in shaping the research that is being directed towards them?

## MODELS OF ENGAGEMENT

Schemes to support the transfer of knowledge from academics to partners outside the university have, in the last decade or so, been largely replaced by an emphasis on knowledge exchange. As Best and Holmes highlight in a social-science policy context, linear models of knowledge transfer have beeen replaced by an emphasis on collaborative relationships and their advocated ‘systems’ approach, which considers the structures and relationships governing interactions with external partners and the potential for change.^30^ In arts and humanities, the AHRC has recently emphasized knowledge exchange in favour of previous knowledge-transfer initiatives.^31^ The differences between these two terms, and indeed the move towards a greater emphasis on two-way exchange rather than one-way transfer, are indicative of a welcome and growing recognition of the importance of interactive engagement. Among arts and humanities researchers, there are a number of different models of engagement, which can often overlap. Firstly, much public engagement rotates around the idea of the academic as an expert who engages and interests a largely passive public. This can take place through the medium of a television or radio broadcast, through academics writing for a lay public, through public talks – and so on. Successful recent examples of the practice include the well-received BBC Radio 4 series, ‘A History of Private Life’, starring historian Amanda Vickery, or the television series ‘Servants: the True Story of Life Downstairs’, which involved Pam Cox and Lucy Delap amongst others. Secondly, historians can act as ‘citizen scholars’. In this model, recently discussed in *History* by John Tosh, academics – particularly historians – can use their knowledge and intellectual expertise to contribute to the democratic society in which they live.^32^ The UK organization History & Policy promotes this approach.^33^ Thirdly, public engagement can involve members of the public as citizen historians, scientists or researchers, able to be more actively involved in academic research and to contribute to collecting and analysing data in some form. This is a model that has been effectively taken up by many academic scientists. Members of the public can contribute to a whole host of scientific projects, and this has become so commonplace that the online encyclopaedia Wikipedia now has a page dedicated to listing them.^34^

Finally, engagement may take the form of more equitable co-production, in which a group of individuals, already in existence or brought together for the purpose, can make more active decisions about what research is undertaken, as well as contributing to the research. Efforts by the AHRC to promote this are very welcome, and the AHRC Connected Communities has provided a number of excellent models for engagement. In part, these echo developments in social sciences more generally, which highlight the benefits of conducting research with and for research subjects. Furthermore, the recently established ‘Knowledge to Action’ model also privileges a close and democratic collaborative relationship with experts and members of the public outside the university.^35^ (One example of this is the Legacies of War project at Leeds, supported in part by AHRC Connected Communities funding, which has worked with a number of community groups and volunteers to examine the history of the First World War in Leeds.) Within this very general aim, some topics of research could be directed, at least in part, by partner organizations who wanted to join the project and by the volunteers who bring their own research interests, agendas and expertise. This idea of engaging people in making history complements rather than displaces other models of engagement fulfilling the same agenda, for example, the expert-transmission model of knowledge exchange. However, while the transmission model elevates scholarly expertise above other forms, our approach fosters a ‘democratic epistemology’. Significantly, democratic epistemology emerges from debates in the USA that do not centre solely on ‘impact’, rather it has developed from a concern with civic politics. In John Saltmarsh and Matthew Hartley’s formulation, it describes a process whereby universities integrate civic engagement into the core of all their activities, and it ‘requires linking the pursuit of knowledge with the pursuit of a healthier society’.^36^ For our purposes, democratic epistemology emphasizes the importance that different kinds of expertise can bring to the process of public engagement and, by extension, expanded opportunities for people to engage with scholarly research, academics and universities. To reorient our understanding of the potential relationships between scholarly research and the public away from impact and towards the ideas of democratic epistemology is to privilege processes of engagement rather than outputs and endpoints. Indeed, when we encourage those we work with to help shape the agenda, our engagement will naturally shift away from our own research interests and publications. The potential of co-production in engagement is huge and exciting, but sits outside current REF definitions of impact. This does not matter in itself, but it poses a threat to the valuing of such activity in institutions which are driven by the vagaries of the REF.

None of these models of public engagement is inherently worse or better than any other. As a pathway to impact, some will function well within the REF context, whereas others will fall beyond what is conceived as impact. Whilst we take the position that the expertise and potential contribution of members of the public is usually underestimated in both the discussion and practice of academics’ engagement, we also recognize that some kind of structure can be effective in permitting a meaningful and valuable engagement, for both sides. Here we can learn from other professions’ approaches and evaluation of public engagement. As Adair, Filene and Koloski note in the context of museums and the heritage sector, ‘whether online, on the streets, in the galleries, or in a recording booth, audiences express themselves more creatively if operating within, not beyond, boundaries’.^37^ The structure provided by academics and universities in engagement can be highly valuable.

By nature, all of these models of public engagement are much more focused on the process of engagement rather than the end-product, though the results cannot be divorced from the engagement itself, whose value often lies in this process, the relationships and collaboration rather than the end change. This is frequently undervalued in the current climate in the UK and beyond.

## OUR CASE STUDIES

Our first case study focuses on a relationship between Laura King and Babakas, an international theatre company based in Birmingham, and their production *Our Fathers*. The relationship started as part of King’s brief to work on public engagement projects as part of a fellowship funded by a Wellcome Trust Strategic Award at the Centre for the History of Medicine, University of Warwick in 2011–12. King’s involvement, according to the company, helped *Our Fathers* develop from a small-scale piece of theatre to a more ambitious production, devised by the company and based on the real lives of these performers. The dramaturg, Brian Mullin, and one of the performers, Mike Tweddle, note how King’s knowledge of the wider context of the history of family life helped ‘universalize’ the stories told in *Our Fathers*, moving it from a tale about three adults, their relationships with their fathers, and their decisions about themselves becoming parents to a much bigger story of social change and intergenerational relationships. Yet it was not only the disseminating of facts and figures of historical research that made this interaction significant but also the discussion of the ideas and concepts involved in the show, the debates about the roles of father and mother over time, and whether parenting was instinctive and unchangeable or historically and culturally determined. Working with the company in genuine collaboration also influenced King’s own research on family life and fatherhood. Considering the emotions and memories involved in family life in past and present, and exploring the way an individual story can relate to the bigger social picture, have informed King’s research and use of testimony as a source. For example, it has strongly influenced the way she has approached oral history interviews with fathers in new research, including the attention paid in these interviews to intergenerational relationships. *Our Fathers* also considers the influence of history itself, on a very personal level, which has again shaped King’s practice as a historian; it has demonstrated for King the importance of considering the influence of the past on a very individualized basis, as well as on a more collective level.

Our second case study, the Stories of Activism in Sheffield, 1960–present, was part of a wider research programme based at the University of Sheffield,^38^ which asked how and why have people become politically actively and under what conditions has this occurred? The project provided its co-founders, neither of them experts in twentieth-century British political or social history, with the opportunity to explore this question in a localized setting. It relied upon a strong working relationship with local activists, who since 2010 have taken the lead in guiding its aims and agenda. The central research question became part of a collaborative process which moved the project far beyond its remit and opened up spaces for activists to start to think about their own pasts and how they should be captured and used. Co-operating academics and non-academics produced a series of principles to define the core elements of the project. Further activities included activist-led workshops, training events and creative collaborations, involving people from a range of social and ethnic backgrounds. The project gave people in Sheffield the opportunity to co-produce knowledge about their past. Spaces and opportunities have emerged for people to recollect, revaluate and recount personal stories in ways previously unavailable to them. In these spaces, discussions about the meanings of activism and how people saw themselves as activists were an important outcome. Furthermore, the practical challenges that activists remember facing drew attention to their everyday problems, for example, the difficulties surrounding the gathering of resources for campaign materials revealed the challenges activists faced when organizing a protest. As a historian of early modern Britain, Rivett has benefited from these conversations, which have helped him to refine his questions about political engagement during the English Civil War. For example, they have raised important questions about the accessibility to the marketplace of print and other essential resources that would have enabled – or, conversely, restricted – how an individual engaged in politics.

These two very different case studies illustrate the exciting and valuable nature of engagement, but also the narrow way in which ‘impact’ is currently defined and valued. In neither project did published research findings have a particular influence; King’s project focused on the exchange of ideas and Rivett’s research on the early modern period did not link directly to the Stories of Activism project. Each was valuable because they transcended the original research in order to involve collaborators in devising the central projects and questions. It was the researcher rather than the research itself that had an influence. Furthermore, in each case value is situated in the process of engagement, the point at which researcher and collaborators come together, rather than in any end product. Finally, the democratic nature of both projects, in which the academic involved was absolutely not in charge and other participants were under no pressure to take up their ideas, meant that for all parties the potential for meaningful exchange was maximized. These crucial aspects would not necessarily be understood as worthwhile within current REF definitions of impact.

## CONCLUSIONS

Our model emphasizes a ‘bottom-up’ approach to engaging with the world beyond the campus, in which publics, partners and academics come together to negotiate the value of different ways of working. We need to return to the ideas at the root of ‘people’s history’, which as Samuel noted, ‘has the merit of raising a crucial question for both theoretical and political work – that of the production of knowledge, both the sources on which it draws and its ultimate point of address’.^39^ Our approach suggests a relational model of how academics’ impact should be valued, in which research is not only disseminated – nor is it the only outcome – but is used to create sustained and mutually beneficial relationships between researchers and members of the public. This would value a less arrogant approach to the question of impact, suggesting that research could have valuable uses outside the academy, but that we should not assume it must be the one and only thing that makes a change. Scholarship may be fundamental to these relationships, or its questions may open spaces of engagement and opportunities for relationships to emerge and grow – often without finding a specific form. Partnerships and collaborations can develop which are horizontal, rather than vertical, in nature. In other words, and in keeping with the premise of ‘democratic epistemology’, knowledge does not flow or transfer from expert to lay member of the public. As in Samuel’s definition of people’s history, ‘[i]t questions the existing intellectual division of labour and implicitly challenges the professionalized monopolies of knowledge’.^40^ To open up our research to partners and publics outside our universities, and to involve people in the creation of different kinds of knowledge, may mean that the process is indeed of more value to the publics and partners involved than to the researcher. Where the dissemination model privileges an academic’s research – which is often near completion or already completed when dissemination occurs – this relational model challenges the conventional hierarchy of experts. As a result and on these terms, research-led engagement is open-ended with an outcome that is not prescribed, and takes seriously the concept of ‘engagement’, relocating the value of this work as about process rather than outcome.^41^ And in this sense of external engagement, it is the arts and humanities that can and do lead the way.

In summary, we suggest that ‘impact’ inappropriately focuses attention on the end product of the relationship of academics with those outside academia. We should not be as paternalistic as to suggest our research is always the answer – academics are not and should not be politicians. A renegotiation of the emphasis on what is valuable in terms of engagement or impact could also address the concerns of academics that the impact agenda in the UK may substantially alter arts and humanities research, marginalizing research that does not directly answer current economic, political or social concerns by loosening the connection between published research and impact and/or engagement. Furthermore, it is often the process of engagement that is inherently most valuable to those we engage with – the conversations, debates and exchanges of skills and ideas rather than the final impact or change. And indeed potential change may come at a much later date, when no measurement will be in place to capture it. Finally, whilst we can do everything to make our research relevant to wider world concerns, by translating it into the right language and selecting appropriate content, by making it accessible, by spending time disseminating results and working with partners to further understanding of what we do, we still cannot control the behaviour of others, and so cannot reliably secure ‘impact’. We can work hard to establish effective pathways to impact, but cannot ensure it. The REF system is in this sense flawed, as it measures what we by definition cannot reliably achieve without some measure of luck or serendipity.

Furthermore, effective impact is most often, in the arts and humanities, achieved in ways outside the current REF definitions. Some of the most effective impact we have as academics is when we work in collaboration with others. To work in genuine collaboration, we must allow a sharing of aims and direction in the making of history, and therefore we cannot impose our research findings on a given group. This may happen with co-produced research: effective co-production will necessitate a move away from the academic researcher(s)’ own research findings. The subsequent process of co-produced historical knowledge usually has a substantial impact on the participants involved (including the researcher) but this does not constitute ‘impact’ under REF definitions because it is not derived from specific research findings. This relates to another core issue: it is often the researcher as a professional and knowledgeable individual who has the impact in an arts and humanities context rather than their actual research conclusions. It is their ability to critique, their skills and their theoretical approach as well as their background knowledge that has most ‘impact’, but again, without a tie to specific research findings, this impact is ineligible in REF definitions. For the future, we hope that a different emphasis in how we understand impact for the purposes of the next REF exercise can bring new energy and potential to the engagement of historians, and other arts and humanities researchers, with the world beyond the campus.

## NOTES AND REFERENCES

1 Raphael Samuel, *Theatres of Memory: Past and Present in Contemporary Culture: vol. 1*, London, 1994, p. 15.

2 Some of the most influential articles have recently been compiled in *The Public History Reader*, ed. Hilda Kean and Paul Martin, London, 2013.

3 A prime example of this is the ‘You Are What You Ate’ project, based on medieval and early modern historical and archaeological expertise: http://www.leeds.ac.uk/youarewhatyouate/ (accessed 22 Dec. 2014).

4 For a discussion of this perspective see Stefan Collini, *What are Universities For?*, London, 2012, pp. 86–111.

5 Pat Thane, ‘History and Policy’, *History Workshop Journal *67, 2009, p. 140.

6 Pam Cox, ‘The Future Uses of History’, *History Workshop Journal *75, 2013.

7 Jo Guldi and David Armitage, *The History Manifesto*, Cambridge, 2014.

8 See Deborah Cohen and Peter Mandler, ‘*The History Manifesto*: a Critique’, *American Historical Review* 120, 2015. See also: http://www.deborahacohen.com/profile/?q=content/critique-history-manifesto (accessed 18 Jan. 2015).

9 For the Connected Communities project see: http://www.ahrc.ac.uk/Funding-Opportunities/Research-funding/Connected-Communities/Pages/Connected-Communities.aspx (accessed 5 Sept. 2013).

10 Available on the NCCPE website: http://www.publicengagement.ac.uk/support-it/manifesto-public-engagement.

11 Raphael Samuel Centre, http://www.raphael-samuel.org.uk/about-centre (accessed 19 Dec. 2014).

12 See ‘Operation Overload’ *Research Fortnight: REF Special*, p. 13. Available at http://exquisitelife.researchresearch.com/files/rf4470.pdf (accessed 19 Dec. 2014).

13 Academics are under pressure to fulfill impact activities as part of their required duties. However, it remains to be seen if universities will credit these activities on the same level as teaching, research and administration when assessing applications for promotion. The time-intensive and ongoing nature of public engagement can also reduce an academic’s time for research, for example, and thereby weaken future chances of promotion.

14 For these figures see: http://www.hefce.ac.uk/news/newsarchive/2011/news62310.html. The document outlining the rationale can be found in *Decisions on Assessing Research Impact*, March 2011, available at http://www.ref.ac.uk/pubs/ (accessed 19 May 2014).

15 Connections exist. As the REF’s 2011 *Decisions on Assessing Research Impact* stated: ‘this [report] reflects policy [the assessment of research impact] aims across the four UK funding bodies to maintain and improve the achievements of the higher education sector, both in undertaking excellent research and in building on this research to achieve demonstrable benefits to the wider economy and society’. See ‘Decisions on Assessing Research Impact’, p. 3: http://www.ref.ac.uk/pubs/2011-01/ (accessed 26 May 2014). The four funding bodies include HEFCE, HEFCW, the Department for Education and Learning and the SFC.

16 ‘RCUK Impact Requirements: Frequently asked questions’: http://www.ahrc.ac.uk/Funding-Opportunities/Documents/RCUKImpactFAQ.pdf (accessed 26 May 2014).

17 For more information, see http://www.ahrc.ac.uk/Funding-Opportunities/Pages/Follow-on-Funding.aspx (accessed 12 Dec. 2014).

18 See ‘Panel Criteria and Working Methods: Assessment Criteria for Panel D’, Section D3, available at http://www.ref.ac.uk/pubs/2012-01/ (accessed 28 May 2014).

19 ‘Panel Criteria and Working Methods’, p. 93.

20 The decision by institutions to imitate successful submission strategies could be one outcome of REF2014. For comments on this issue see Chris O’Brien, ‘REF 2014 results and the future of “impact”’, *Bulletin*: http://www.bulletin.co.uk/24176/ref-2014-results-future-impact/ (accessed 19 Dec. 2014)

21 For more information, see http://www.ahrc.ac.uk/Funding-Opportunities/Research-funding/Connected-Communities/Pages/HLF-All-Our-Stories-Initiative.aspx. The Legacies of War project at Leeds (http://arts.leeds.ac.uk/legaciesofwar/themes/yorkshire-and-the-great-war/) provides further examples of this.

22 ‘Panel Criteria and Working Methods’, Section D3, paragraph 101b.

23 Rhys Jones, Jessica Pykett and Mark Whitehead, ‘The Geographies of Soft Paternalism in the UK: the Rise of the Avuncular State and Changing Behaviour after Neoliberalism’, *Geography Compass* 5: 1, 2011, p. 51.

24 Richard H. Thaler and Cass R. Sunstein, *Nudge: Improving Decisions about Health, Wealth and Happiness*, London, 2008.

25 *Concordat for Engaging the Public with Research, *RCUK: http://www.rcuk.ac.uk/Publications/policy/perConcordat/ (accessed 26 May 2014).

26 RCUK, ‘Inspiration to Engage: Concordat for Engaging the Public with Research’, p. 6: http://www.rcuk.ac.uk/publications/reports/inspiration/ (accessed 26 May 2014).

27 RCUK, ‘Inspiration to Engage’, p. 15.

28 Peter Mandler, ‘While you were Looking Elsewhere … the Haldane Principle and the Government’s Research Agenda for the Arts and Humanities’, http://www.nacbs.org/blog/opinion-peter-mandler-while-you-were-looking-elsewherethe-haldane-principle-and-the-governments-research-agenda-for-the-arts-and-humanities (accessed 25 June 2014); James Vernon, ‘The State They Are In’, http://www.historians.org/publications-and-directories/perspectives-on-history/march-2011/the-state-they-are-in-history-and-public-education-in-england (accessed 24 June 2014).

29 Collini, *What Are Universities For?*, p. 175.

30 Allan Best and Bev Holmes, ‘Systems Thinking, Knowledge and Action: towards Better Models and Methods’, *The Policy Press* 6: 2, 2010.

31 The AHRC previously published its Knowledge Transfer Strategy 2008–11, whereas the term is not used in its Delivery Plan 2011–15. The AHRC now runs a Knowledge Transfer Partnership scheme, but by and large the emphasis is on Knowledge Exchange. See AHRC Knowledge Transfer Strategy 2008–11: http://www.ahrc.ac.uk/news-and-events/publications/documents/knowledge-transfer-strategy-2008-2011.pdf; AHRC Delivery Plan 201115: http://www.ahrc.ac.uk/News-and-Events/Publications/Documents/Delivery-Plan-2011-2015.pdf (all accessed 16 Jan. 2015).

32 John Tosh, ‘Public History, Civic Engagement and the Historical Profession in Britain’, *History* 99: 335, 2014.

33 See http://www.historyandpolicy.org (accessed 22 Dec. 14).

34 http://en.wikipedia.org/wiki/List_of_citizen_science_projects (accessed 29 May 2013).

35 On Knowledge to Action models, see Best and Holmes, ‘Systems Thinking’, pp. 145–59; Bren Neale and Sarah Morton, ‘Creating Impact Through Qualitative Longitudinal Research’, *Timescapes Methods Guides Series* 20. For an example of a current project, Following Young Fathers, see http://followingfathers.leeds.ac.uk (accessed 22 Jan. 2015).

36 John Saltmarsh and Matthew Hartley, ‘To Serve a Larger Purpose’, in ‘“*To Serve a Larger Purpose”: Engagement for Democracy and the Transformation of Higher Education*, ed. Saltmarsh and Hartley, Philadelphia, 2011, pp. 3–4. Discussions in the USA are particularly advanced on the issues under discussion here. See for example, James Quay and James Veninga, ‘Making Connections: the Humanities, Culture and the Community’, *National Task Force on Scholarship and the Public Humanities*, New York, 1990. More recently, see *Practising Public Scholarship: Experiences and Possibilities Beyond the Academy*, ed. Katharyne Mitchell, Hoboken NJ, 2008.

37 Bill Adair, Benjamin Filene and Laura Koloski, ‘Introduction’, in *Letting Go? Sharing Historical Authority in a User-Generated World*, ed. Adair, Filene and Koloski, Philadelphia, 2011, p. 12. Also see Kevin Walker, ‘Structuring Visitor Participation’, in *Digital Technologies and the Museum Experience*: *Handheld Guides and Other Media*, ed. Loic Tallon and Kevin Walker, Alta Mira, 2008.

38 The larger research programme was a Leverhulme Trust-funded research network, entitled *The Comparative History of Political Engagement in Western and African Societies*, based at the University of Sheffield. For more details of the network see: http://www.historyofpoliticalengagement.dept.shef.ac.uk.

39 Raphael Samuel, ‘People’s History’, in Samuel, *People’s History and Socialist Theory*, London, 1981, p. xxxii.

40 As previous note.

41 There are similarities between our approach and that outlined in Martin Bashforth and Patricia Bashforth, ‘Diverse Evill Persons: Echoes in the Landscape, Echoes in the Archives’, *Public History Review* 18, 2011, see esp. p. 104. In relation to their project at Cannon Hall, Bashforth and Bashforth state: ‘The aim is to create a space within which the visitor can bring their own experience and influences into an unpredictable play with the material presented by the historian and the multi-sense experiences provided by the artist, to create open-ended opportunities for the creation and sharing of new meanings that neither party expected’.

